# Prehospital diagnostic algorithm for acute coronary syndrome using machine learning: a prospective observational study

**DOI:** 10.1038/s41598-022-18650-6

**Published:** 2022-08-26

**Authors:** Masahiko Takeda, Takehiko Oami, Yosuke Hayashi, Tadanaga Shimada, Noriyuki Hattori, Kazuya Tateishi, Rie E. Miura, Yasuo Yamao, Ryuzo Abe, Yoshio Kobayashi, Taka-aki Nakada

**Affiliations:** 1 Department of Emergency and Critical Care Medicine, Chiba University Graduate School of Medicine, 1-8-1 Inohana, Chuo, Chiba, 260-8677 Japan; 2 Department of Cardiovascular Medicine, Chiba University Graduate School of Medicine, Chiba, Japan; 3 Smart119 Inc., 7th floor, Chiba Chuo Twin Building No. 2, 2-5-1 Chuo, Chiba, Japan

**Keywords:** Cardiology, Acute coronary syndromes, Medical research

## Abstract

Rapid and precise prehospital recognition of acute coronary syndrome (ACS) is key to improving clinical outcomes. The aim of this study was to investigate a predictive power for predicting ACS using the machine learning-based prehospital algorithm. We conducted a multicenter observational prospective study that included 10 participating facilities in an urban area of Japan. The data from consecutive adult patients, identified by emergency medical service personnel with suspected ACS, were analyzed. In this study, we used nested cross-validation to evaluate the predictive performance of the model. The primary outcomes were binary classification models for ACS prediction based on the nine machine learning algorithms. The voting classifier model for ACS using 43 features had the highest area under the receiver operating curve (AUC) (0.861 [95% CI 0.775–0.832]) in the test score. After validating the accuracy of the model using the external cohort, we repeated the analysis with a limited number of selected features. The performance of the algorithms using 17 features remained high AUC (voting classifier, 0.864 [95% CI 0.830–0.898], support vector machine (radial basis function), 0.864 [95% CI 0.829–0.887]) in the test score. We found that the machine learning-based prehospital algorithms showed a high predictive power for predicting ACS.

## Introduction

Early therapeutic interventions are crucial for reducing the mortality of acute coronary syndrome (ACS)^1^. A substantial number of patients have initial symptoms of ACS outside hospitals; emergency medical service (EMS) personnel play a role as the first responders to patients. EMS personnel estimate the possibility of ACS based on the symptoms of patients and transport them to the appropriate hospital for immediate treatment. Precise prediction of ACS in the prehospital setting may contribute to improving the quality of ACS care and clinical outcomes.

Several studies have investigated the prediction of ACS. Integrated components of patient history, vital signs, 12-lead electrocardiograms (ECG), and cardiac enzymes were studied to increase the accuracy of diagnosis in prehospital management^2^. Prehospital 12-lead ECG is recommended for early diagnosis in patients with suspected ST-segment elevation myocardial infarction (STEMI)^3^; however, costs and lack of training of 12-lead ECG limit its widespread use^4,5^. Other diagnostic tools with cardiac biomarkers have demonstrated efficacy for risk stratification, but several concerns, including technical errors, high false-negative rates, and possible delays in transportation, cast a shadow on the generalization of promising results^6^.

As a result of the low utility of 12-lead ECG and biochemical tests in the prehospital setting, a novel diagnostic tool with vital signs, 3-lead ECG monitoring, and symptoms is warranted to improve the diagnostic accuracy of EMS personnel. Optimized prehospital system interventions in the field of stroke potentially reduce treatment delays and improve clinical outcomes^7,8^. With the development of machine learning approaches, early prediction models for stroke have demonstrated their accurate and stable performance^9^. However, there are few studies using machine learning to predict the onset of ACS in a prehospital setting.

Therefore, the aim of this study was to evaluate a predictive power of the machine learning algorithms predicting ACS based on vital signs, 3-lead ECG monitoring, and symptoms using a large cohort of patients with suspected ACS.

## Results

### Baseline characteristics and outcomes

After a series of exclusions, 555 patients were included in the internal cohort, 192 (35%) patients were diagnosed with ACS (Table 1). Of the 61 patients included in the external cohort, 29 (48%) patients were diagnosed with ACS (Supplemental Table S1). ACS patients had significantly lower age, a higher proportion of males, lower frequency of stable angina, lower heart rate, lower body temperature, higher blood oxygen saturation, and higher frequency of ST elevation or ST change than non-ACS patients. For the symptoms, ACS patients had greater pain severity and higher proportion of cold hands, hand moistening, pressing pain, nausea or vomiting, cold sweat, pain radiating to jaw or shoulder, and persistent pain than non-ACS patients. In the external cohort, ACS patients had significantly lower age, lower heart rate, higher frequency of ST elevation or ST change than non-ACS patients, which was consistent with the internal cohort.

**Table 1:** Baseline characteristics and clinical outcomes in the internal cohort.

	ACS (n = 192)	Non-ACS (n = 363)	P value
Age, years	68 (58.5–77)	73 (60–82)	0.005
Male sex, n (%)	152 (79.2)	214 (59.0)	< 0.001
**Past medical history**			
Diabetes mellitus, n (%)	37 (19.3)	63 (17.4)	0.577
Hypertension, n (%)	72 (37.5)	146 (40.2)	0.532
Dyslipidemia, n (%)	14 (7.3)	17 (4.7)	0.203
Stable angina, n (%)	15 (7.8)	60 (16.5)	0.004
Old myocardial infarction, n (%)	24 (12.5)	66 (18.2)	0.084
Prior PCI, n (%)	19 (9.9)	45 (12.4)	0.380
Prior CABG, n (%)	2 (1.0)	6 (1.7)	0.566
Intracranial hemorrhage, n (%)	2 (1.0)	3 (0.8)	0.799
Cerebral infarction, n (%)	11 (5.7)	20 (5.5)	0.915
Prior antiplatelet or anticoagulant therapy, n (%)	12 (6.3)	40 (11.0)	0.067
**Vital signs**			
Heart rate (beats/min)	74 (60–90)	88 (72–110)	< 0.001
Systolic blood pressure (mmHg)	143 (120–169)	147.5 (122–176)	0.237
Diastolic blood pressure (mmHg)	87 (70–102)	87.5 (72–102)	0.926
Body temperature (°C)	36.0 (35.8–36.2)	36.2 (36.0–36.8)	< 0.001
Blood oxygen saturation (%)	98 (96–99)	97 (93–99)	< 0.001
Respiratory rate (times/min)	20 (18–24)	20 (18–24)	0.007
Japan Coma Scale = 0, n (%)	167 (87.0)	293 (80.7)	0.062
Oxygen therapy, n (%)	69 (35.9)	143 (39.4)	0.425
**ECG monitoring**			
ST elevation, n (%)	94 (49.0)	28 (7.7)	< 0.001
ST depression, n (%)	62 (32.3)	115 (31.7)	0.883
ST change, n (%)	156 (81.3)	143 (39.4)	< 0.001
Arrhythmia, n (%)	44 (22.9)	84 (23.1)	0.953
**Symptoms**			
1. Cold hands, n (%)	76 (39.6)	89 (24.5)	< 0.001
2. Hand moistening, n (%)	66 (34.4)	79 (21.7)	0.001
3. Dyspnea, n (%)	47 (24.5)	121 (33.3)	0.031
4. Palpitations, n (%)	30 (15.6)	97 (26.7)	0.003
5. Throbbing pain, n (%)	40 (20.8)	72 (19.8)	0.780
6. Sharp/stabbing pain, n (%)	17 (8.9)	25 (6.9)	0.405
7. Positional chest pain, n (%)	25 (13.0)	33 (9.1)	0.150
8. Reproduction of chest pain by palpation, n (%)	6 (3.1)	11 (3.0)	0.951
9. Chest pain with breathing or cough, n (%)	7 (3.7)	20 (5.5)	0.332
10. Pressing pain, n (%)	149 (77.6)	226 (62.3)	< 0.001
11. Nausea or vomiting, n (%)	54 (28.1)	55 (15.2)	< 0.001
12. Cold sweat, n (%)	111 (57.8)	117 (32.2)	< 0.001
13. Pain radiating to jaw or shoulder, n (%)	31 (16.2)	21 (5.8)	< 0.001
14. Similarity to previous ischemic episode, n (%)	29 (15.1)	68 (18.7)	0.284
15. Chest pain aggravated by walk, n (%)	22 (11.5)	53 (14.6)	0.303
16. Worsening pain, n (%)	52 (27.1)	99 (27.3)	0.962
17. Pain at rest, n (%)	151 (78.7)	262 (72.2)	0.097
18. Persistent pain, n (%)	179 (93.2)	291 (80.2)	< 0.001
19. Recurrent pain within 24 h, n (%)	41 (21.4)	64 (17.6)	0.287
20. Chronic pain, n (%)	16 (8.3)	58 (16.0)	0.012
21. Pain severity (10-point scale)	6 (0–8)	4 (0–7)	0.005

Data are presented as median and interquartile range for continuous features.

*P-*values were calculated using Pearson’s chi-square test or Mann–Whitney U test.

CABG (coronary artery bypass grafting), ECG (electrocardiogram), PCI (percutaneous coronary intervention).

### Prediction of ACS

The Voting classifier model, which was comprised of all machine learning algorithms used in this study for the prediction of ACS using 43 features, showed the highest area under the receiver operating characteristic curve (AUC) (0.861 [95% CI 0.775–0.832]) in the test score (Table 2). The eXtreme Gradient Boosting (XGBoost) model for the onset of ACS using 43 features showed the highest predictive power (AUC 0.839 [95% CI 0.734–0.931]) in the external cohort score (Table 2).

**Table 2:** Prehospital diagnostic algorithms for acute coronary syndrome using 43 features.

Models	AUC	Sensitivity	Accuracy	Specificity	F1-score	PPV	NPV
**Training score**							
XGBoost	0.887	0.819	0.887	0.826	0.755	0.712	0.890
Logistic regression	0.893	0.818	0.893	0.807	0.762	0.698	0.906
Random forest	0.922	0.860	0.922	0.874	0.805	0.781	0.909
SVM (Linear)	0.894	0.822	0.894	0.823	0.762	0.713	0.898
SVM (radial basis function)	0.902	0.842	0.902	0.836	0.790	0.737	0.916
MLP	0.893	0.829	0.893	0.826	0.772	0.722	0.905
LDA	0.890	0.826	0.890	0.834	0.763	0.723	0.894
LGBM	0.894	0.823	0.894	0.819	0.764	0.711	0.902
Voting	0.927	0.852	0.927	0.839	0.804	0.744	0.928
**Test score**							
XGBoost	0.849	0.756	0.792	0.811	0.715	0.684	0.864
Random forest	0.850	0.755	0.798	0.821	0.725	0.711	0.865
Logistic regression	0.843	0.740	0.780	0.801	0.703	0.693	0.857
SVM (Linear)	0.847	0.745	0.789	0.813	0.709	0.690	0.861
SVM (radial basis function)	0.834	0.735	0.791	0.821	0.708	0.687	0.855
MLP	0.834	0.709	0.786	0.826	0.695	0.695	0.846
LDA	0.860	0.761	0.802	0.823	0.727	0.706	0.870
LGBM	0.841	0.756	0.778	0.791	0.705	0.671	0.860
Voting	0.861	0.772	0.803	0.821	0.733	0.711	0.873
**External cohort score**							
XGBoost	0.840	0.897	0.790	0.697	0.800	0.722	0.885
Random forest	0.803	0.690	0.726	0.758	0.702	0.714	0.735
Logistic regression	0.831	0.793	0.758	0.727	0.754	0.719	0.800
SVM (Linear)	0.838	0.793	0.758	0.727	0.754	0.719	0.800
SVM (radial basis function)	0.808	0.828	0.742	0.667	0.750	0.686	0.815
MLP	0.818	0.793	0.758	0.727	0.754	0.719	0.800
LDA	0.832	0.862	0.774	0.697	0.781	0.714	0.852
LGBM	0.789	0.552	0.742	0.909	0.667	0.842	0.698
Voting	0.828	0.862	0.790	0.727	0.794	0.735	0.857

AUC (area under the receiver operating characteristic curve), LDA (linear discriminant analysis), LGBM (light gradient boosting machine), MLP (multilayer perceptron), NPV (negative predictive values), PPV (posititive predictive values), SVM (support vector machine), XGBoost (eXtreme Gradient Boosting).

### Feature selection for the prediction algorithm

**Figure 1:**
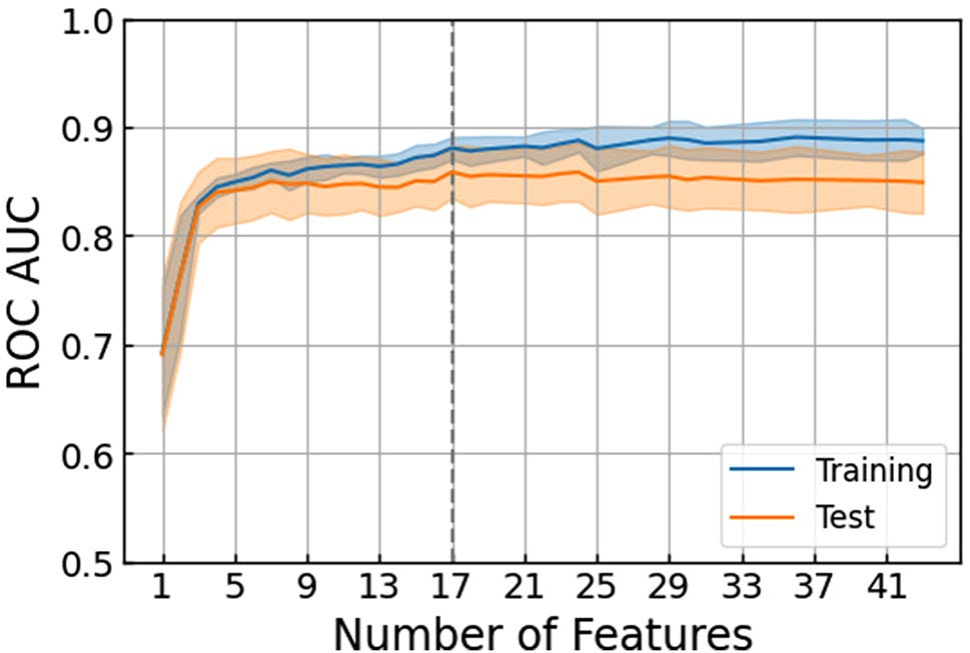
Relationship between the number of features and the area under the receiver operating characteristic curve for the prediction algorithm. The line plot depicts sequential changes in the AUC with the number of features for the prediction algorithm in (**a**) the training score (blue) and (**b**) the test score (yellow). The dotted vertical line indicates the highest predictive value in the test score. (n = 17, AUC of the training score = 0.881, AUC of the test score = 0.859). The error bars indicate 95% confidence intervals. AUC (area under the receiver operating characteristic curve).

**Figure 2:**
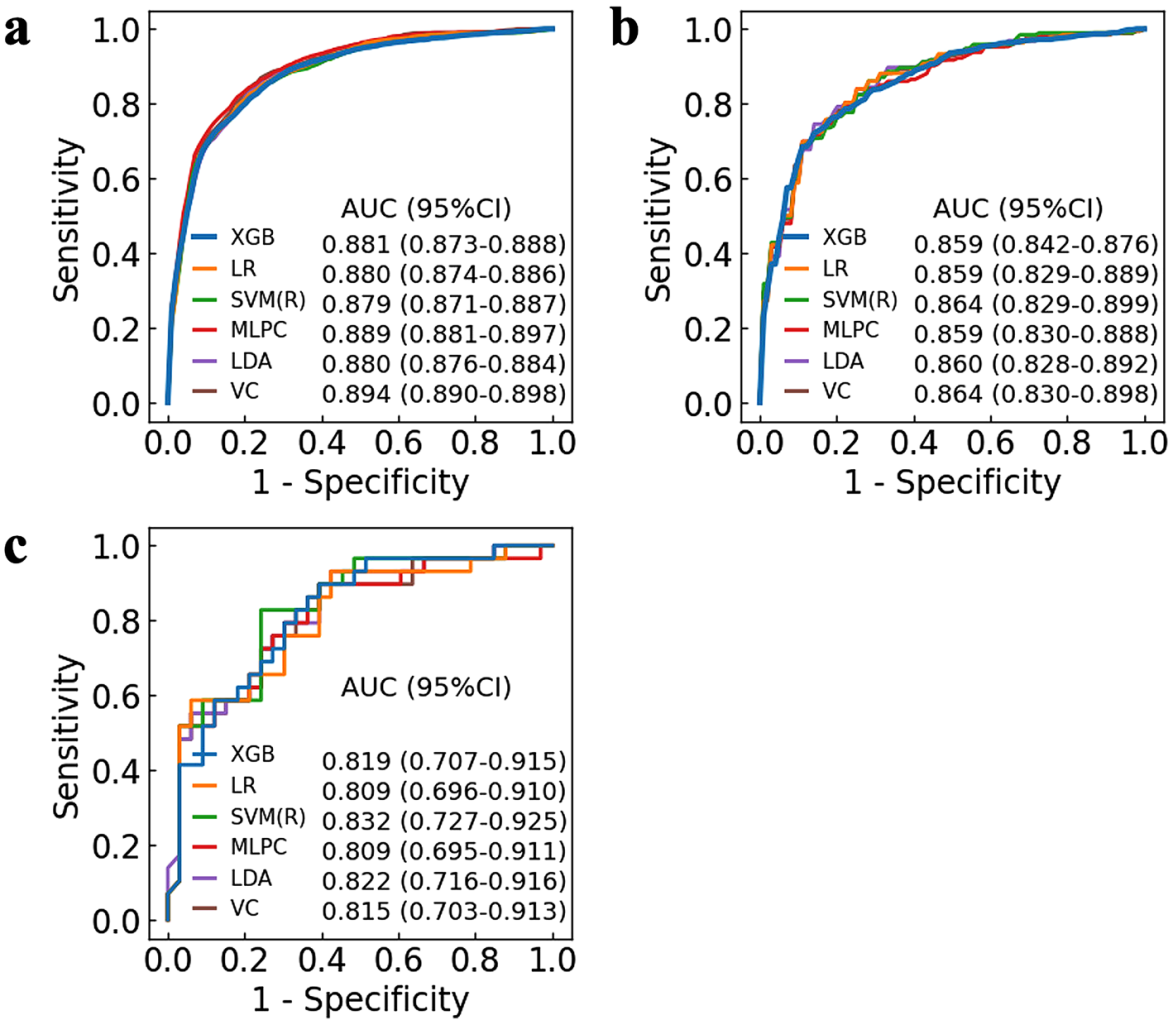
Receiver operating characteristic curve of prehospital diagnostic algorithms for acute coronary syndrome with 17 features. ROC curves of the top six machine learning algorithms for the prehospital prediction of ACS using 17 features are shown. The ROC curves are depicted at 1-specificity on the x-axis and sensitivity on the y-axis using (**a**) the training score, (**b**) the test score, and (**c**) external cohort score. AUC is presented with 95% confidence interval. ACS (acute coronary syndrome), AUC (area under the receiver operating characteristic curve), CI (confidence interval), LDA (linear discriminant analysis), LR (logistic regression), MLPC (multilayer perceptron classifier), ROC (receiver operating characteristic), SVM (R) (support vector machine radial basis function), VC (voting classifier), XGB (eXtreme Gradient Boosting).

We examined the relationship between the number of features and the change in predictive values, including AUC, accuracy, sensitivity, specificity, F1-score, PPV (positive predictive values), and NPV (negative predictive values), using XGBoost (Fig. 1 and Supplementary Fig. S1). While reducing the number of features from 43 to 17, the AUC remained high in the test score (17 features 0.859 [95% CI 0.842–0.876], 43 features 0.849 [95% CI 0.772–0.812]) (Table 2, Supplementary Table S2, and Supplementary Fig. S2). However, in decreasing the number of features from 16 to 1, the prediction algorithm with fewer features had lower predictive values. Of the nine machine learning algorithms with 17 features, the voting classifiers model and the support vector machine (SVM) (radial basis function) model had the highest predictive value (voting classifier, AUC 0.864 [95% CI 0.830–0.898], SVM (radial basis function), AUC 0.864 [95% CI 0.829–0.899]) in the test score (Fig. 2 and Supplementary Table S2). The SVM (radial basis function) model for ACS using 17 features showed the highest AUC (0.832 [95% CI 0.727–0.925]) in the external cohort score (Supplementary Table S2).

**Figure 3:**
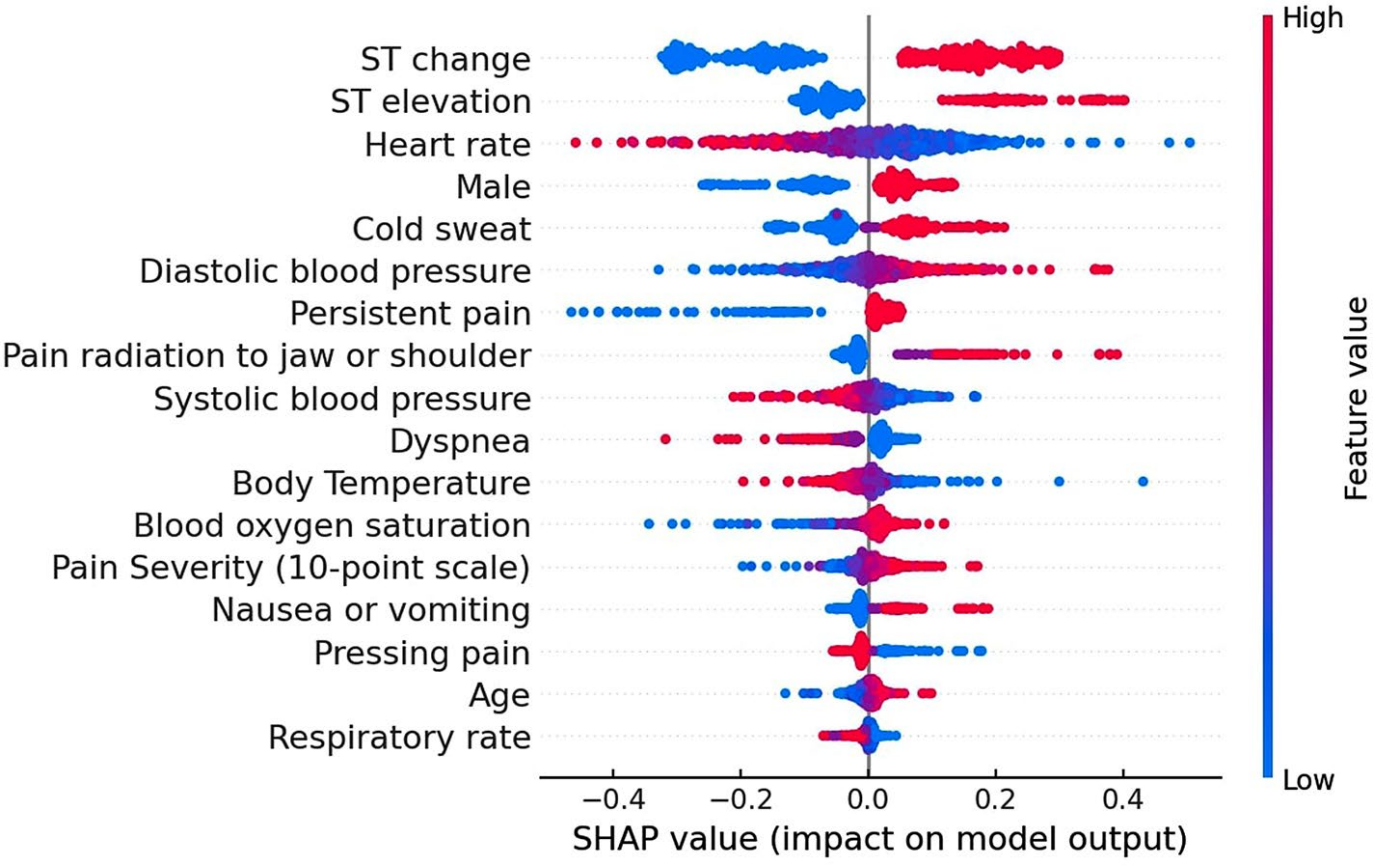
SHAP values of the prehospital diagnostic algorithm for acute coronary syndrome using 17 features. The impact of the features on the model output was expressed as the SHAP value calculated with the support vector machine (radial basis function). The features are placed in descending order according to their importance. The association between the feature value and SHAP value indicates a positive or negative impact of the predictors. The extent of the value is depicted as red (high) or blue (low) plots. SHAP (SHapley Additive exPlanation).

The SHAP values of the prehospital diagnostic algorithm for ACS using 43 and 17 features were calculated with the linear discriminant analysis (Supplementary Fig. S3) and the SVM (radial basis function) (Fig. 3), respectively. The SHAP summary plot revealed that “ST change,” “ST elevation,” and “heart rate” were particularly important predictors of ACS, followed by “cold sweat” and “male”.

### Prediction of AMI or STEMI

Next, we built classification models for diagnosing subcategories of ACS, including acute myocardial infarction (AMI) and ST-segment elevation myocardial infarction (STEMI), using the nine machine learning algorithms with 17 features. The prediction algorithms of AMI using the SVM (linear) model and the multilayer perceptron (MLP) model also had the highest predictive value (SVM (linear), AUC 0.850 [95% CI 0.817–0.884], MLP, AUC 0.850 [95% CI 0.817–0.882]) in the test score (Supplementary Table S3). The linear discriminant analysis (LDA) model presented the highest AUC for the prediction of STEMI (0.862 [95% CI 0.831–0.894]) in the test score (Supplementary Table S4).

## Discussion

In this study, we found that the machine learning-based prehospital model showed a high predictive power for predicting the diagnosis of ACS and subcategories of ACS using 17 features including vital signs, 3-lead ECG monitoring, and symptoms. This accurate diagnostic algorithm may contribute to early prediction of diagnosis in prehospital settings and reduce the transport time to a facility where therapeutic intervention is available, even without special equipment or technical training.

Although machine learning-based prediction algorithms have shown promising results with high accuracy in other fields, including stroke and acute aortic syndrome^9,10^, to the best of our knowledge, only one study has reported the efficacy of a machine learning-based prediction model for the prehospital onset of ACS using only 12-lead ECG^11^. In contrast, in our study, we built the models on the basis of 3-lead ECG monitoring, as well as vital signs and symptoms, which can be easily obtained without special equipment and technical training in a prehospital setting. The strength of this study is the remarkably high predictive values of our machine learning models, even when the model inputs are limited to easily obtainable features. Our voting classifier model for the prediction of ACS using 17 features model showed a superior predictive power (AUC = 0.864 in the test score) compared to the previously reported models using 12-lead ECG (AUC = 0.82)^11^. Furthermore, compared to the widely used standard scoring system (HEART score: AUC = 0.84) for patients with suspected ACS in the emergency department^11^, our models had a higher predictive power even in the prehospital setting.

While several studies have demonstrated the efficacy and feasibility of risk stratification for ACS with combined modalities such as 12-lead ECG and biomarkers in the emergency department^12–14^ and prehospital setting^2^, there are few reports predicting the onset of ACS according to vital signs, ECG monitoring, and symptoms obtained by EMS personnel. A prehospital stroke scale with physical examination has been^15^ designed to be accessible and applicable for EMS personnel initially triaging patients with limited information, but the conventional scoring system for suspected ACS requires 12-lead ECG and cardiac troponin in addition to medical history^13^. A previous study^16^, which compared diagnostic accuracy for ACS between an assessment of general practitioners and clinical decision rule (CDR) based on medical history and physical examination, reported that the AUC was 0.66 for the physicians’ risk estimate and 0.75 for the CDR. This result implies that the diagnostic precision for ACS based on physical assessment reaches the ceiling when 12-lead ECG or cardiac enzymes are not available. In this context, our novel approach for predicting the onset of ACS with vital signs, ECG monitoring, and symptoms using machine learning would provide us with substantial advantages over traditional methods.

With the high predictive accuracy of the algorithm for the diagnosis of ACS, the SHAP analysis presented significant features contributing to the diagnosis of ACS: ST change, ST elevation, heart rate, cold sweat, and male. While 12-lead ECG has been recognized as one of the most reliable tests for estimating the probability of diagnosis, ECG monitoring with leads I, II, or III demonstrated noteworthy findings for an assessment of the likelihood. Other features listed as contributing factors are potentially used as additional information to determine the possibility of ACS in a prehospital setting. Based on the extent of the contribution to the diagnosis, we successfully decreased the number of features for the prediction algorithm from 43 to 17 features. This can be explained by that the exclusion of the irrelevant and redundant features, and noises has improved the model performance. The advantages of the modified algorithm with a decreased number of features include reduction of workload and shorter duration of implementation, leading to potential feasibility of clinical application in the future. Such a diagnostic tool with a predicting algorithm is soon to be launched with validation in a prehospital setting.

Some limitations of this study need to be addressed. First, the specific study area, Chiba city, could be an obstacle for generalization of the results, although the study was conducted in multiple institutions. Second, patient background such as dyslipidemia in our study is different from that in previous studies^17^. Insufficient interviews with a limited time may be attributed to missing information. Third, in this study, the 663 screened patients, 108 (16%) were excluded, which could have led to selection bias. The most common reason for the exclusion was missing diagnostic data, which was due to insufficient or delayed data entry at each site. As the data are publicly available, the objective analysis would enhance the robustness. Fourth, the proportion of patients with STEMI in this study (83%) is higher than that in the Japanese registry data (approximately 70%)^18^. Selection bias is a potential reason for the lower percentage of patients with NSTEMI and UA. Fifth, the prediction algorithm for diagnosing NSTEMI was not developed in the analysis because of the lack of sufficient data. As ECG shows low sensitivity in NSTEMI^19,20^, our algorithm estimating the probability of ACS could improve the diagnostic accuracy of NSTEMI. Future studies should clarify the predictive value of NSTEMI, as well as the robustness of diagnostic accuracy for STEMI using the algorithm. Sixth, we used 3-lead ECG monitoring to determine ECG changes. Although few studies have directly compared 3-lead ECG monitoring with 12-lead ECG, sufficient performance of 3-lead ECG for the prediction of ACS has been reported in the situation where 12-lead ECG is unavailable^21^. While 12-lead ECG may have a better predictive power, machine learning algorithms based on promptly available 3-lead ECG monitoring, vital signs, and symptoms showed a high predictive power.

In conclusion, we found that the prehospital prediction algorithm had a high predictive power for diagnosing the onset of ACS using machine learning from the data of vital signs, 3-lead ECG monitoring, and symptoms obtained by EMS personnel. Further investigations are needed to validate the accuracy and feasibility of the algorithm in a prehospital setting.

## Methods

### Study population

This study was a multicenter observational study that was prospectively conducted in an urban area of Japan (Chiba city, population 1 million). Enrolled patients from September 1, 2018 to March 5, 2021 and from March 6, 2021 to April 27, 2022 were assigned to the internal cohort and the external cohort, respectively. Consecutive adult patients (≥ 20 years of age) identified by EMS personnel with suspected ACS who were transported to one of the twelve participating facilities were enrolled in the study. The symptoms indicating ACS to EMS personnel included pain, discomfort, or pressure in the chest, epigastric region, neck, jaw, or shoulder within 24 h. Patients with other symptoms that were strongly suspected of having an onset of ACS were also enrolled in the study. Patients with cardiac arrest were excluded from the study because they could not be interviewed in a manner consistent with the other patients.

The study was approved by the Ethical Review Board of the Graduate School of Medicine, Chiba University (No. 2733). In accordance with the Ethical Guidelines for Medical and Health Research Involving Human Subjects in Japan, the requirement for written informed consent was waived by the review board.

### Data collection and definition

We collected data from 663 patients in the internal cohort for 45 features used to predict ACS in a prehospital setting. These features included past medical history, vital signs, 3-lead ECG monitoring, and 21 symptoms (Supplementary Table S5). However, we used only 43 features after excluding two low variance features that were constant in more than 95% of the sample, specifically, the past medical histories of “Prior coronary artery bypass grafting (CABG)” and “Intracranial hemorrhage.” The onset timing and meteorological conditions were considered, but discarded in the final analysis (see Supplementary Note S1 for contribution of onset timing and meteorological conditions).

ST changes were assessed with leads I, II, or III of ECG monitoring. ST changes included ST elevation and ST depression. Assessment of the ST changes were left to the discretion of EMS personnel. The contents of symptoms were determined based on previous studies^12–14,22,23^. Symptoms 1 and 2 were evaluated by palpation, and symptoms 3–21 were evaluated via interviews. Detailed interview data are shown in Supplementary Table S6. The diagnosis of ACS was established by cardiologists with findings from a catheter angiography according to current guidelines^24^. ACS was defined as acute myocardial infarction (AMI) and unstable angina (UA).

Of the 663 screened patients in the internal cohort, 555 patients were included in the final analysis after excluding 108 patients because of missing diagnostic data, multiple entries, and cardiac arrest (Supplementary Fig. S4). Of the 69 screened patients in the external cohort, 61 patients were included in the final analysis after exclusion of 8 patients due to missing diagnostic data and multiple entries.

### Missing values

As our data had missing values for some features, we performed imputations before building the machine learning models. We used the imputed values as input even to the gradient boosting model, which can deal with missing values by treating them the same way as categorical values, because we found that our imputation approach written below had improved its performance compared to the implementation without imputation. Following the domain knowledge, we mutually imputed the missing values in some features: symptoms 4 to 21, except symptoms 19 and 20, and a pair of systolic and diastolic blood pressure. The vital signs, including body temperature, blood oxygen saturation, and breathing rate, were imputed with each median value. For any other categorical attribute, the missing values were replaced with a new subcategory “Unknown”.

### Machine learning model development

In this study, we used nested cross-validation to evaluate the predictive performance of the model, because the nested cross-validation procedure produces robust and unbiased performance estimates regardless of sample size^25–27^(see Supplementary Note S2 for detailed descriptions of our nested cross-validation).

First, we developed binary classification models for ACS prediction as a primary outcome based on nine machine leaning algorithms: XGBoost, logistic regression, random forest, SVM (linear), SVM (radial basis function), MLP, LDA, light gradient boosting machine (LGBM) classifier and voting classifier comprised of all machine learning used in this study. For the selection of machine learning, a popular method was chosen with reference to previous reports^28,29^. The voting classifier was selected as an ensemble method of all the rest of classifiers above. As a secondary outcome, we built binary classification models for AMI and STEMI prediction. Non-ST-segment elevation myocardial infarction (NSTEMI) was not included in the secondary analysis because of its small number. The parameters were optimized using the grid search method with nested cross-validation.

We assessed the feature importance in the machine learning model based on the Shapley Additive exPlanation (SHAP) value^30^, which was calculated using the machine learning algorithms with the highest AUC in the test score. The voting classifier was excluded from the algorithms to calculate the SHAP values due to the lack of available code. The SHAP value is a solution concept used in game theory and is computed by the difference in model output resulting from the inclusion of a feature in the algorithm, providing information on the impact of each feature on the output. The SHAP value is a method for its interpretability in machine learning models and is also used as a feature selection tool. A higher absolute SHAP value indicates a more important feature.

### Feature selections

We also performed feature selection by discarding the redundant and irrelevant features for prediction to improve performance and the interpretability of the model using XGBoost. We used XGBoost for feature selections because the algorithm handles both linear and nonlinear data and missing data efficiently and flexibly. Also, the accuracy of the algorithm is stable even in the analysis with redundant variables^31^. Feature selection was performed by the following steps: i.e., (1) We built models using 42 features with dropping one feature from 43 features and evaluated the model through nested CV (5-outer fold and 5-outer fold). (2) We replaced the feature to be removed with another feature and repeated this for 43 features. (3) The best combinations of the explainable feature were selected by ROC AUC of these 43 models. (4) The procedures (1)–(3) were repeated until the number of features became one. This process was repeated 10 times to avoid less important features appearing in the higher ranking by chance. As a result of the iterations, we determined the most plausible number of features (i.e., the most important features to be included) from the model that showed the best performance in the mean CV scores. After feature selection, we built a classification model for ACS prediction using nine machine leaning algorithms with the 17 selected features.

## Statistical analysis

We expressed the data as median (interquartile range) values for continuous variables and absolute numbers and percentages for categorical variables. The model performance was evaluated using AUC, accuracy, sensitivity, specificity, F1 score, PPV and NPV. Statistical significance was set at *P* < 0.05. We used Python 3.7.13 packages (NumPy 1.21.6, Pandas 1.1.5, XGBoost 1.4.0, and Scikit-learn 1.0.2) to construct the machine learning models and Prism (version 7.0, GraphPad Software, San Diego, CA) for statistical analysis. The code and data for the analysis of this study are available online (https://github.com/rm119/prehospital_diagnostic_algorithm_for_acute_coronary_syndrome_using_machine_learning).

## Supplementary Information

Supplementary Information.

## Data Availability

The datasets used and analyzed during our study are available from the corresponding author upon reasonable request.

## References

[1] Anderson JL, Morrow DA (2017). Acute myocardial infarction. N. Engl. J. Med..

[2] Knoery CR (2020). Systematic review of clinical decision support systems for prehospital acute coronary syndrome identification. Crit. Pathw. Cardiol..

[3] Welsford M (2015). Part 5: Acute coronary syndromes: 2015 international consensus on cardiopulmonary resuscitation and emergency cardiovascular care science with treatment recommendations. Circulation.

[4] Kimura K (2019). JCS 2018 guideline on diagnosis and treatment of acute coronary syndrome. Circ. J..

[5] Mori H (2021). The impact of pre-hospital 12-lead electrocardiogram and first contact by cardiologist in patients with ST-elevation myocardial infarction in Kanagawa, Japan. J. Cardiol..

[6] van Dongen DN (2020). Pre-hospital risk assessment in suspected non-ST-elevation acute coronary syndrome: A prospective observational study. Eur. Heart J. Acute Cardiovasc. Care.

[7] Chowdhury SZ, Baskar PS, Bhaskar S (2021). Effect of prehospital workflow optimization on treatment delays and clinical outcomes in acute ischemic stroke: A systematic review and meta-analysis. Acad. Emerg. Med..

[8] Chowdhury SZ (2021). Optimising prehospital pathways to improve acute stroke reperfusion therapy delivery: Systems-based approaches. SN Compr. Clin. Med..

[9] Suzuki K (2018). Emergent large vessel occlusion screen is an ideal prehospital scale to avoid missing endovascular therapy in acute stroke. Stroke.

[10] Duceau B (2020). Prehospital triage of acute aortic syndrome using a machine learning algorithm. Br. J. Surg..

[11] Al-Zaiti S (2020). Machine learning-based prediction of acute coronary syndrome using only the pre-hospital 12-lead electrocardiogram. Nat. Commun..

[12] Antman EM (2000). The TIMI risk score for unstable angina/non-ST elevation MI: A method for prognostication and therapeutic decision making. JAMA.

[13] Six AJ, Backus BE, Kelder JC (2008). Chest pain in the emergency room: Value of the HEART score. Neth. Heart J..

[14] Fox KA (2006). Prediction of risk of death and myocardial infarction in the six months after presentation with acute coronary syndrome: Prospective multinational observational study (GRACE). BMJ.

[15] Kothari RU, Pancioli A, Liu T, Brott T, Broderick J (1999). Cincinnati prehospital stroke scale: Reproducibility and validity. Ann. Emerg. Med..

[16] Bruins Slot MH (2011). Diagnosing acute coronary syndrome in primary care: Comparison of the physicians’ risk estimation and a clinical decision rule. Fam. Pract..

[17] Ando H (2022). Japanese nationwide PCI (J-PCI) registry annual report 2019: Patient demographics and in-hospital outcomes. Cardiovasc. Interv. Ther..

[18] Ishihara M (2015). Clinical presentation, management and outcome of japanese patients with acute myocardial infarction in the troponin era - Japanese registry of acute myocardial infarction diagnosed by universal definition (J-MINUET). Circ. J..

[19] Collet JP (2021). 2020 ESC guidelines for the management of acute coronary syndromes in patients presenting without persistent ST-segment elevation. Eur. Heart J..

[20] Amsterdam EA (2014). 2014 AHA/ACC guideline for the management of patients with non-ST-elevation acute coronary syndromes: A report of the American college of cardiology/American heart association task force on practice guidelines. J. Am. Coll. Cardiol..

[21] Green M (2007). Best leads in the standard electrocardiogram for the emergency detection of acute coronary syndrome. J. Electrocardiol..

[22] Swap CJ, Nagurney JT (2005). Value and limitations of chest pain history in the evaluation of patients with suspected acute coronary syndromes. JAMA.

[23] Morrow DA (2000). TIMI risk score for ST-elevation myocardial infarction: A convenient, bedside, clinical score for risk assessment at presentation: An intravenous nPA for treatment of infarcting myocardium early II trial substudy. Circulation.

[24] Thygesen K (2018). Fourth universal definition of myocardial infarction (2018). J. Am. Coll. Cardiol..

[25] Krstajic D, Buturovic LJ, Leahy DE, Thomas S (2014). Cross-validation pitfalls when selecting and assessing regression and classification models. J. Cheminformatics.

[26] Varma S, Simon R (2006). Bias in error estimation when using cross-validation for model selection. BMC Bioinform..

[27] Vabalas A, Gowen E, Poliakoff E, Casson AJ (2019). Machine learning algorithm validation with a limited sample size. PLoS ONE.

[28] Stewart J (2021). Applications of machine learning to undifferentiated chest pain in the emergency department: A systematic review. PLoS ONE.

[29] Deo RC (2015). Machine learning in medicine. Circulation.

[30] Lundberg SM (2020). From local explanations to global understanding with explainable AI for trees. Nat. Mach. Intell..

[31] Hou N (2020). Predicting 30-days mortality for MIMIC-III patients with sepsis-3: A machine learning approach using XGboost. J. Transl. Med..

