# Supplementary material for: Prehospital diagnostic algorithm for acute coronary syndrome using machine learning: a prospective observational study
Source: Sci Rep. 2022 Aug 26;12:14593. doi: 10.1038/s41598-022-18650-6 (PMC9418242; doi:10.1038/s41598-022-18650-6)
Supplement: Supplementary file 1 — Supplementary Information. [file 41598_2022_18650_MOESM1_ESM.pdf]

Supplementary Material for

Prehospital diagnostic algorithm for acute coronary syndrome using machine learning: a prospective observational study

Information

Supplementary Table S1. Baseline characteristics and clinical outcomes of the external cohort

Supplementary Table S2. Prehospital diagnostic algorithms for acute coronary syndrome using 17 features

Supplementary Table S3. Prehospital diagnostic algorithms for acute myocardial infarction using 17 features

Supplementary Table S4. Prehospital diagnostic algorithms for ST-segment elevation myocardial infarction using 17 features

Supplementary Table S5. Collected features

Supplementary Table S6. Interviews of symptoms

Supplementary Figure S1. Relationship between the number of features and accuracy, sensitivity, specificity, F1-score, positive predictive values and negative predictive values for the prediction algorithm

Supplementary Figure S2. Comparison of test score of area under the receiver operating characteristic curve using XGBoost with 17 and 43 features

Supplementary Figure S3. SHAP value of the prehospital diagnostic algorithm for acute coronary syndrome using 43 features

Supplementary Figure S4. Study flowchart

Supplementary Note S1. Contribution of timing and meteorological factors to the onset of acute coronary syndrome

Supplementary Note S2. Model evaluation strategy

Supplementary Table S1. Baseline characteristics and clinical outcomes of the external cohort

	ACS (n=29)	Non-ACS (n=32)	*P* value
Age, years	69 (57-18)	79.5 (67-86)	0.049
Male sex, n (%)	20 (69.0)	19 (59.4)	0.436
Past medical history			
Diabetes mellitus, n (%)	14 (48.3)	5 (15.6)	0.006
Hypertension, n (%)	12 (41.4)	9 (28.1)	0.277
Dyslipidemia, n (%)	1 (3.5)	0 (0.0)	0.290
Stable angina, n (%)	4 (13.8)	7 (21.9)	0.412
Old myocardial infarction, n (%)	3 (10.3)	5 (15.6)	0.542
Prior PCI, n (%)	3 (10.3)	6 (18.8)	0.355
Prior CABG, n (%)	0 (0.0)	0 (0.0)	
Intracranial hemorrhage n (%)	0 (0.0)	0 (0.0)	
Cerebral infarction, n (%)	2 (6.9)	2 (6.3)	0.919
Prior antiplatelet or anticoagulant therapy, n (%)	1 (3.5)	5 (15.6)	0.111
Vital signs			
Heart rate (beats/min)	76 (64-90)	96.5 (78-122)	0.004
Systolic blood pressure (mmHg)	140 (119-155)	160 (134-181)	0.034
Diastolic blood pressure (mmHg)	87 (71-100)	90 (71-106)	0.573
Body temperature (°C)	36.3 (35.8-36.8)	36.4 (36.0-36.6)	0.623
Blood oxygen saturation (%)	99 (96-99)	97 (91-99)	0.007
Respiratory rate (times/min)	24 (18-24)	24 (18-24)	0.662
Japan Coma Scale=0, n (%)	25 (86.2)	27 (84.4)	0.840
Oxygen therapy, n (%)	11 (37.9)	9 (28.1)	0.415
ECG monitoring			
ST elevation, n (%)	14 (48.3)	1 (3.1)	<0.001
ST depression, n (%)	8 (27.6)	12 (37.5)	0.410
ST change, n (%)	22 (75.9)	13 (40.6)	0.005
Arrhythmia, n (%)	3 (10.3)	9 (28.1)	0.001
Symptoms			
1. Cold hands, n (%)	8(27.6)	5 (15.6)	0.255
2. Hand moistening, n (%)	8 (27.6)	7 (21.9)	0.605
3. Dyspnea, n (%)	2 (6.9)	2 (6.3)	0.919
4. Palpitations, n (%)	3 (10.3)	7 (21.9)	0.224
5. Throbbing pain, n (%)	7 (24.1)	7 (21.9)	0.834
6. Sharp/stabbing pain, n (%)	1 (3.5)	1 (3.1)	0.944
7. Positional chest pain, n (%)	3 (10.3)	4 (12.5)	0.792
8. Reproduction of chest pain by palpation, n (%)	0 (0.0)	1 (3.1)	0.337
9. Chest pain with breathing or cough, n (%)	1 (3.5)	2 (6.3)	0.613
10. Pressing pain, n (%)	22 (75.9)	17 (53.1)	0.065
11. Nausea or vomiting, n (%)	8 (27.6)	4 (12.5)	0.139
12. Cold sweat, n (%)	14 (48.3)	11 (34.4)	0.270
13. Pain radiating to jaw or shoulder, n (%)	6 (20.7)	3 (9.4)	0.213
14. Similarity to previous ischemic episode, n (%)	4 (13.8)	5 (15.6)	0.840
15. Chest pain aggravated by walk, n (%)	4 (13.8)	2 (6.3)	0.323
16. Worsening pain, n (%)	8 (27.6)	10 (31.3)	0.754

17. Pain at rest, n (%)	23 (79.3)	26 (81.3)	0.849
18. Persistent pain, n (%)	27 (93.1)	29 (90.6)	0.725
19. Recurrent pain within 24 hours, n (%)	6 (20.7)	5 (15.6)	0.607
20. Chronic pain, n (%)	4 (13.8)	2 (6.3)	0.323
21. Pain severity (10-point scale)	5 (0-7)	0 (0.5-6.5)	0.541

Data are presented as median and interquartile range for continuous features.
P-values were calculated using Pearson's chi-square test or Mann–Whitney U test.
CABG (coronary artery bypass grafting), ECG (electrocardiogram), PCI (percutaneous coronary intervention)

Supplementary Table S2. Prehospital diagnostic algorithms for acute coronary syndrome using 17 features

Models	AUC	Accuracy	Sensitivity	Specificity	F1-score	PPV	NPV
**Training score**							
XGBoost	0.881	0.814	0.795	0.824	0.747	0.709	0.885
Logistic regression	0.880	0.818	0.784	0.836	0.748	0.722	0.881
Random forest	0.924	0.858	0.842	0.867	0.805	0.773	0.912
SVM (Linear)	0.882	0.812	0.800	0.819	0.747	0.704	0.886
SVM (radial basis function)	0.879	0.809	0.814	0.807	0.747	0.691	0.892
MLP	0.889	0.826	0.794	0.843	0.759	0.733	0.887
LDA	0.880	0.800	0.838	0.780	0.744	0.670	0.901
LGBM	0.916	0.840	0.855	0.833	0.788	0.732	0.916
Voting	0.894	0.811	0.845	0.793	0.756	0.685	0.907
**Test score**							
XGBoost	0.859	0.810	0.749	0.842	0.731	0.723	0.866
Random forest	0.853	0.802	0.766	0.821	0.727	0.700	0.871
Logistic regression	0.859	0.805	0.782	0.818	0.737	0.708	0.879
SVM (Linear)	0.858	0.800	0.782	0.810	0.731	0.696	0.878
SVM (radial basis function)	0.864	0.804	0.782	0.816	0.737	0.708	0.877
MLP	0.859	0.820	0.746	0.859	0.743	0.759	0.869
LDA	0.860	0.814	0.772	0.838	0.741	0.724	0.878
LGBM	0.836	0.778	0.772	0.783	0.711	0.671	0.866
Voting	0.864	0.807	0.783	0.821	0.739	0.718	0.881
**External cohort score**							
XGBoost	0.806	0.726	0.655	0.788	0.691	0.731	0.722
Random forest	0.804	0.726	0.759	0.697	0.721	0.688	0.767
Logistic regression	0.809	0.758	0.552	0.939	0.681	0.889	0.705
SVM (Linear)	0.815	0.726	0.655	0.788	0.691	0.731	0.722
SVM (radial basis function)	0.832	0.774	0.793	0.758	0.767	0.742	0.806
MLP	0.809	0.726	0.759	0.697	0.721	0.688	0.767
LDA	0.822	0.726	0.759	0.697	0.721	0.688	0.767
LGBM	0.785	0.710	0.759	0.667	0.710	0.667	0.759
Voting	0.810	0.742	0.828	0.667	0.750	0.686	0.815

AUC (area under the receiver operating characteristic curve), LDA (linear discriminant analysis), LGBM (light gradient boosting machine), MLP (multilayer perceptron), NPV (negative predictive values), PPV (positive predictive values), SVM (support vector machine), XGBoost (eXtreme Gradient Boosting)

Supplementary Table S3. Prehospital diagnostic algorithms for acute myocardial infarction using 17 features

Models	AUC	Accuracy	Sensitivity	Specificity	F1-score	PPV	NPV
Training score							
XGBoost	0.872	0.811	0.775	0.827	0.724	0.684	0.888
Logistic regression	0.870	0.800	0.798	0.801	0.719	0.657	0.896
Random forest	0.901	0.828	0.838	0.824	0.758	0.694	0.916
SVM (Linear)	0.869	0.790	0.815	0.778	0.712	0.635	0.901
SVM (radial basis function)	0.871	0.798	0.807	0.793	0.718	0.648	0.898
MLP	0.875	0.812	0.783	0.825	0.727	0.684	0.892
LDA	0.867	0.791	0.815	0.779	0.713	0.636	0.901
LGBM	0.895	0.821	0.832	0.816	0.749	0.685	0.913
Voting	0.879	0.793	0.847	0.767	0.723	0.631	0.915
**Test score**							
XGBoost	0.841	0.796	0.745	0.820	0.705	0.683	0.873
Random forest	0.845	0.796	0.768	0.810	0.713	0.686	0.882
Logistic regression	0.845	0.784	0.774	0.789	0.700	0.657	0.884
SVM (Linear)	0.850	0.796	0.751	0.818	0.705	0.676	0.876
SVM (radial basis function)	0.849	0.795	0.779	0.802	0.714	0.674	0.888
MLP	0.850	0.789	0.768	0.799	0.700	0.655	0.883
LDA	0.838	0.793	0.746	0.815	0.706	0.692	0.872
LGBM	0.822	0.777	0.748	0.789	0.683	0.651	0.876
Voting	0.848	0.787	0.785	0.789	0.708	0.660	0.889
**External cohort score**							
XGBoost	0.816	0.758	0.828	0.697	0.762	0.706	0.821
Random forest	0.807	0.742	0.724	0.758	0.724	0.724	0.758
Logistic regression	0.806	0.726	0.862	0.606	0.746	0.658	0.833
SVM (Linear)	0.809	0.710	0.828	0.606	0.727	0.649	0.800
SVM (radial basis function)	0.794	0.774	0.552	0.970	0.696	0.941	0.711
MLP	0.834	0.758	0.759	0.758	0.746	0.733	0.781
LDA	0.828	0.742	0.897	0.606	0.765	0.667	0.870
LGBM	0.779	0.710	0.655	0.758	0.679	0.704	0.714
Voting	0.806	0.758	0.552	0.939	0.681	0.889	0.705

AUC (area under the receiver operating characteristic curve), LDA (linear discriminant analysis), LGBM (light gradient boosting machine), MLP (multilayer perceptron), NPV (negative predictive values), PPV (positive predictive values), SVM (support vector machine), XGBoost (eXtreme Gradient Boosting)

Supplementary Table S4. Prehospital diagnostic algorithms for ST-segment elevation myocardial infarction using 17 features

Models	AUC	Accuracy	Sensitivity	Specificity	F1-score	PPV	NPV
**Training score**							
XGBoost	0.891	0.818	0.818	0.822	0.723	0.650	0.919
Logistic regression	0.878	0.793	0.793	0.816	0.706	0.642	0.909
Random forest	0.916	0.843	0.843	0.857	0.767	0.704	0.932
SVM (Linear)	0.878	0.789	0.789	0.819	0.706	0.645	0.908
SVM (radial basis function)	0.876	0.810	0.810	0.807	0.708	0.632	0.914
MLP	0.879	0.774	0.774	0.835	0.709	0.656	0.902
LDA	0.874	0.787	0.787	0.803	0.692	0.620	0.904
LGBM	0.907	0.839	0.839	0.830	0.742	0.665	0.928
Voting	0.880	0.813	0.813	0.795	0.700	0.616	0.914
**Test score**							
XGBoost	0.850	0.755	0.755	0.803	0.676	0.632	0.893
Random forest	0.857	0.742	0.742	0.838	0.698	0.668	0.889
Logistic regression	0.852	0.724	0.724	0.844	0.688	0.675	0.886
SVM (Linear)	0.855	0.736	0.736	0.843	0.698	0.683	0.891
SVM (radial basis function)	0.851	0.736	0.736	0.818	0.679	0.650	0.887
MLP	0.854	0.768	0.768	0.806	0.685	0.626	0.897
LDA	0.862	0.761	0.761	0.803	0.682	0.641	0.895
LGBM	0.841	0.698	0.698	0.846	0.672	0.674	0.877
Voting	0.856	0.755	0.755	0.813	0.684	0.642	0.893
**External cohort score**							
XGBoost	0.784	0.739	0.739	0.692	0.654	0.586	0.818
Random forest	0.785	0.783	0.783	0.718	0.692	0.621	0.848
Logistic regression	0.814	0.565	0.565	0.949	0.684	0.867	0.787
SVM (Linear)	0.805	0.565	0.565	0.923	0.667	0.813	0.783
SVM (radial basis function)	0.781	0.565	0.565	0.897	0.650	0.765	0.778
MLP	0.804	0.565	0.565	0.949	0.684	0.867	0.787
LDA	0.823	0.783	0.783	0.718	0.692	0.621	0.848
LGBM	0.794	0.696	0.696	0.718	0.640	0.593	0.800
Voting	0.821	0.783	0.783	0.718	0.692	0.621	0.848

AUC (area under the receiver operating characteristic curve), LDA (linear discriminant analysis), LGBM (light gradient boosting machine), MLP (multilayer perceptron), NPV (negative predictive values), PPV (positive predictive values), SVM (support vector machine), XGBoost (eXtreme Gradient Boosting)

Supplementary Table S5. Collected features

Age	Symptoms
Sex	1. Cold hands
	2. Hand moistening
Past medical history	3. Dyspnea
Diabetes mellitus	4. Palpitations
Hypertension	5. Throbbing pain
Dyslipidemia	6. Sharp/stabbing pain
Stable angina	7. Positional chest pain
Old myocardial infarction	8. Reproduction of chest pain by palpation
Prior PCI	9. Chest pain with breathing or cough
Prior CABG	10. Pressing pain
Intracranial hemorrhage	11. Nausea or vomiting
Cerebral infarction	12. Cold sweat
Prior antiplatelet or anticoagulant therapy	13. Radiation to jaw or shoulder
	14. Similarity to previous ischemic episode
Vital signs	15. Chest pain aggravated by walk
Heart rate	16. Worsening pain
Systolic blood pressure	17. Pain at rest
Diastolic blood pressure	18. Persistent pain
Body temperature	19. Recurrent pain with 24 hours
Blood oxygen saturation	20. Chronic pain
Respiratory rate	21. Pain severity
Japan Coma Scale	
Oxygen therapy	
ECG monitoring	
ST elevation	
ST depression	
ST change	
Arrhythmia	

CABG (coronary artery bypass grafting), ECG (electrocardiogram), PCI (percutaneous coronary intervention)

Supplementary Table S6. Interviews of symptoms

Element	Question
3. Dyspnea	Do you have difficulty in breathing?
4. Palpitations	Do you have palpitations?
5. Throbbing pain	Do you describe the pain as throbbing?
6. Sharp/stabbing pain	Do you describe the pain as sharp or stabbing?
7. Positional chest pain	Does the pain get better or worse when you change your body position?
8. Reproduction of chest pain by palpation	If I touch your chest wall, does it reproduce the pain?
9. Chest pain with breathing or cough	Does the pain get worse when you take a deep breath or cough?
10. Pressing pain	Do you describe the pain as heavy or pressing?
11. Nausea or vomiting	Do you have nausea? Did you vomit?
12. Cold sweat	Do you have cold sweat?
13. Pain radiating to jaw or shoulder	Does your pain radiate to your jaw or shoulder?
14. Similarity to previous ischemic episode	If you have experienced a heart attack or angina in the past, is this pain similar to the pain you had then?
15. Chest pain aggravated by walk	Does the pain get worse when you walk?
16. Worsening pain	Is the pain getting worse since it began?
17. Pain at rest	Do you have the pain at rest?
18. Persistent pain	Is the pain still present?
19. Recurrent pain with 24 hours	Have you had the pain more than once in the past 24 hours?
20. Chronic pain	Do you have the pain for more than 6 months?
21. Pain severity	If 10 is the most severe pain you have ever had, on the 10-point scale, how severe is this pain?

Supplementary Figure S1. Relationship between the number of features and accuracy, sensitivity, specificity, F1-score, positive predictive values and negative predictive values for the prediction algorithm

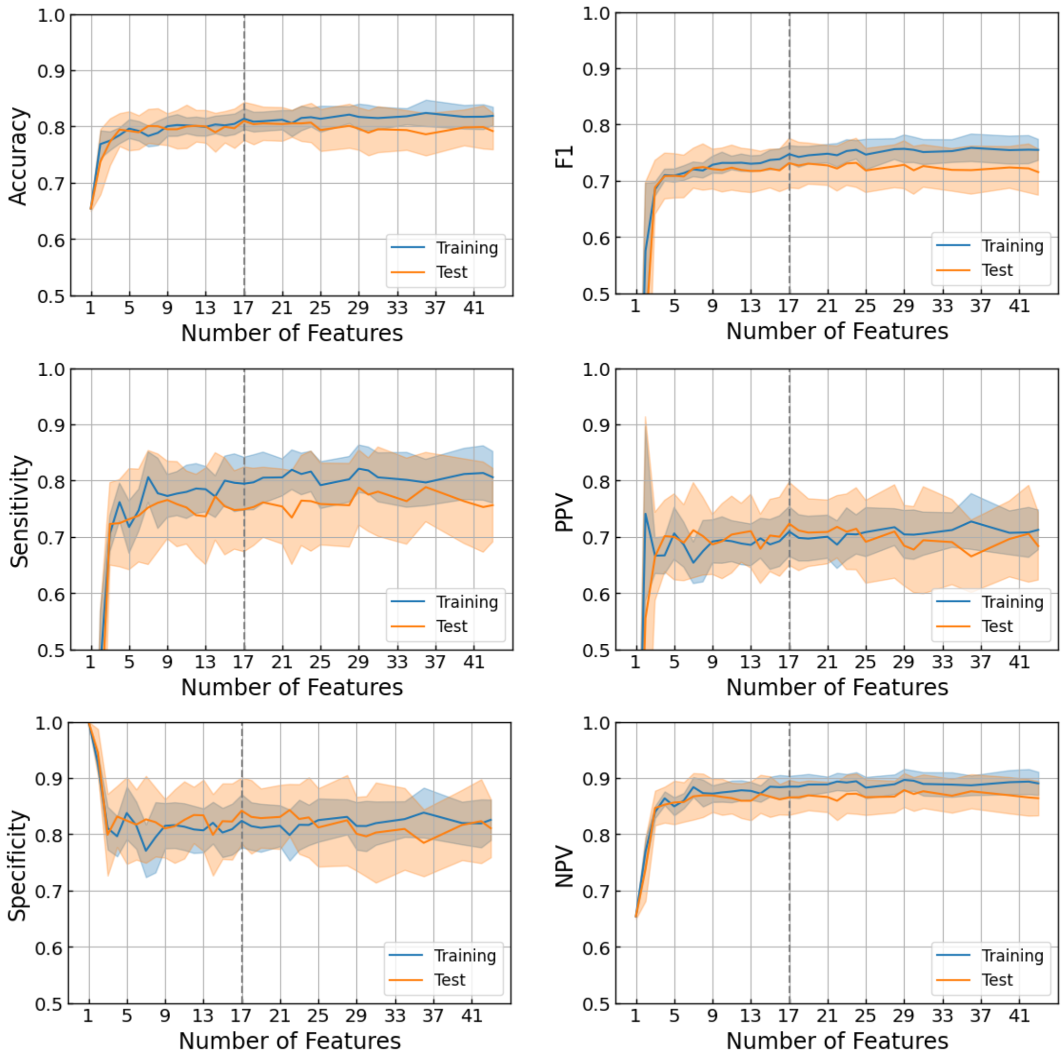

The line plots depict sequential changes in the accuracy, sensitivity, specificity, F1-score, positive predictive values, and negative predictive values with the number of features for the prediction algorithm (a) in the training score (blue) and (b) the test score (yellow). The dotted vertical line indicates n=17. These 17 features produced the highest average accuracy (training score 0.814, test score 0.810) and positive predictive values (training score 0.709, test score 0.723) in the test score. The error bars indicate 95% confidence intervals.

NPV (negative predictive values), PPV (positive predictive values)

Supplementary Figure S2. Comparison of test score of area under the receiver operating characteristic curve using XGBoost with 17 and 43 features

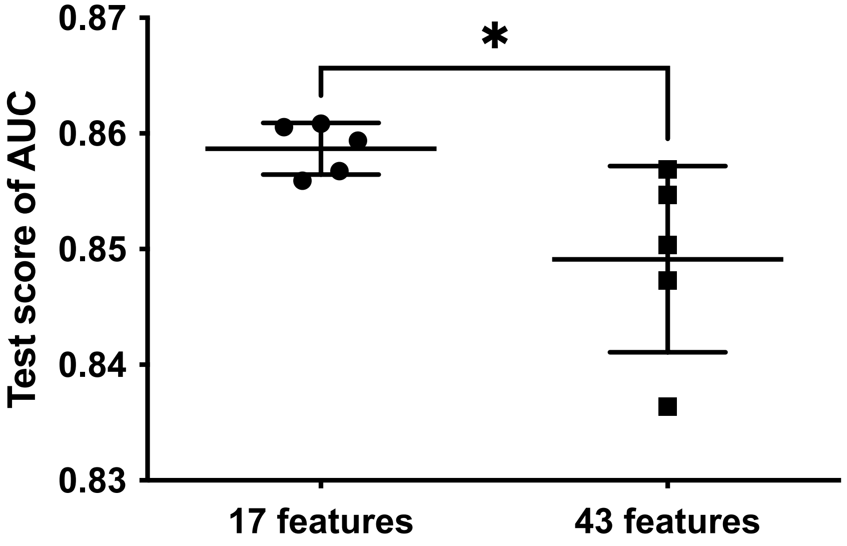

The average test score of the AUC were calculated for each session. Then, the mean test score was compared between XGBoost with 17 and 43 features. XGBoost using 17 features showed a significantly higher test score (0.859 [95% CI 0.842–0.876]) than that using 43 features (0.849 [95% CI 0.772–0.812]) (P = 0.034). *: P = 0.034 using an unpaired *t*-test. AUC (area under the receiver operating characteristic curve), XGBoost (eXtreme Gradient Boosting), CI (confidence interval).

Supplementary Figure S3. SHAP value of the prehospital diagnostic algorithm for acute coronary syndrome using 43 features

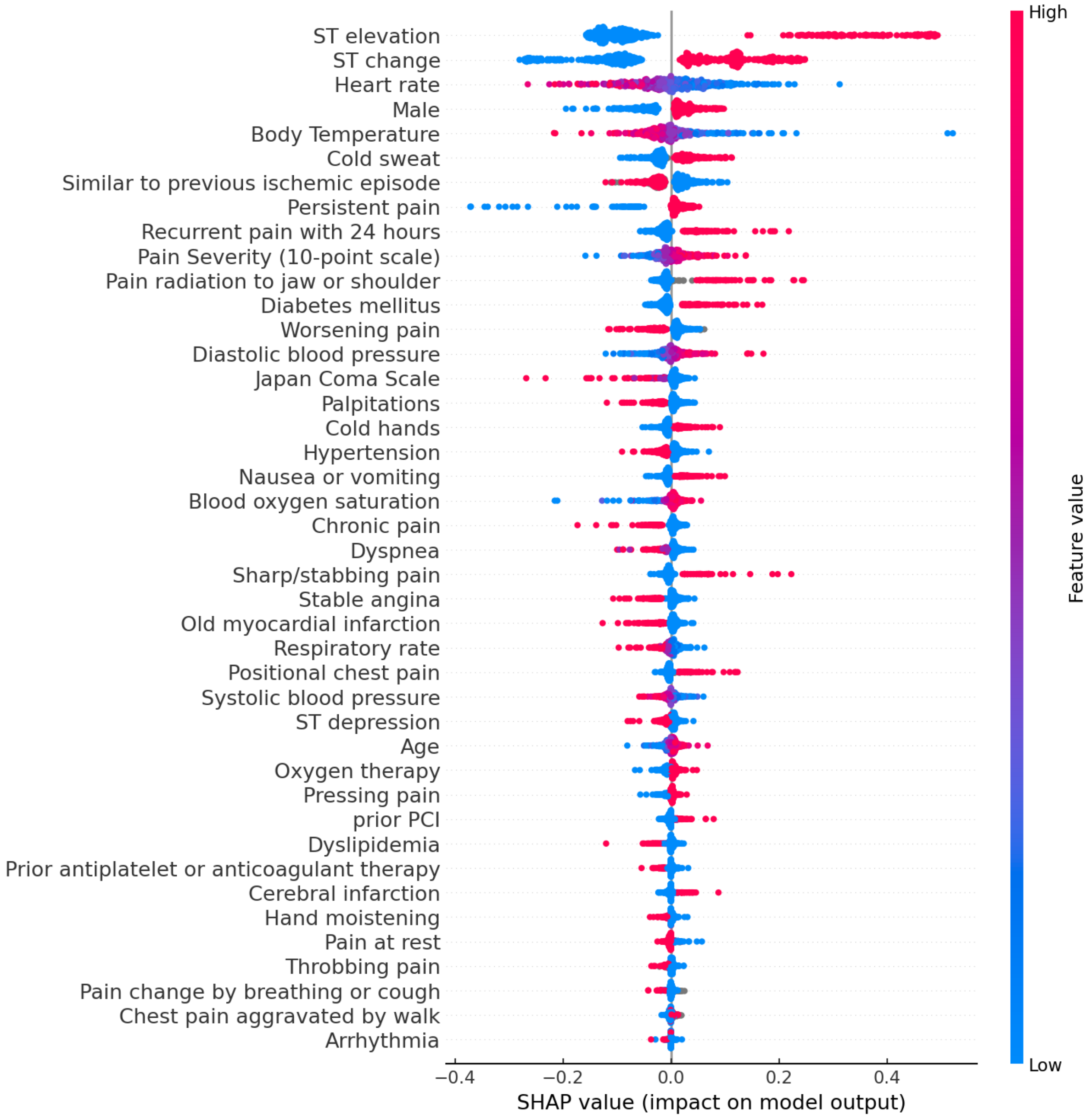

The impact of the features on the model output is expressed as the SHAP value calculated with the linear discriminant analysis. The features are placed in descending order according to their importance. The association between the feature value and SHAP value indicates a positive or negative impact of the predictors. The extent of this value is depicted in red (high) or blue (low). SHAP (SHapley Additive exPlanation).

Supplementary Figure S4. Study flowchart

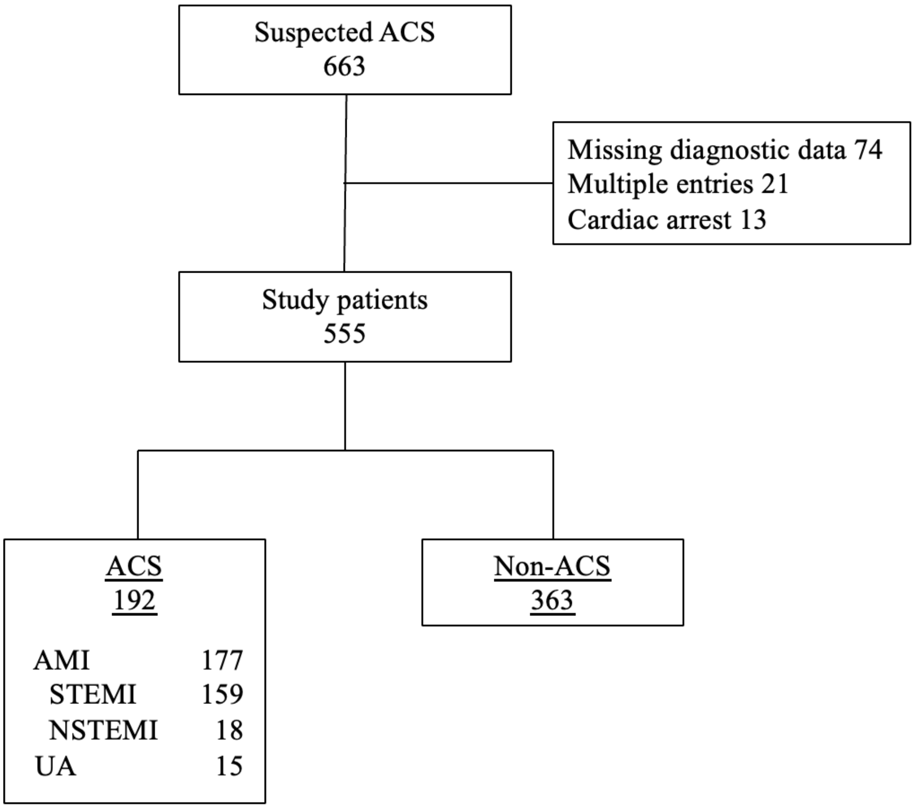

ACS (acute coronary syndrome), AMI (acute myocardial infarction), NSTEMI (non- ST-segment elevation myocardial infarction), STEMI (ST-segment elevation myocardial infarction), UA (unstable angina).

Supplementary Note S1. Contribution of timing and meteorological conditions to the onset of acute coronary syndrome

The onset timing (hours, weekday) and meteorological conditions on the onset day were also considered as important feature candidates as suggested in previous studies [1-3]. However, none of these features were selected by our feature selection method or by another method based on statistical correlation. This might be due to an insufficient number of samples compared to the wide range of hours and weekdays in our data; therefore, we leave this for future analysis.

Supplementary Note S2. Model evaluation strategy

We used nested cross-validation (CV) with 5-outer folds and 5-inner folds to fine-tune the model parameters and to select the best combination of the explainable features, because the nested CV procedure produces robust and unbiased performance estimates regardless of sample size [4,5].

Nested CV includes a double loop, an outer loop, and an inner loop to avoid data leakage. In the nested CV, the training cohort was split into five subsets, leaving one subset for model evaluation and the remaining four subsets for model training (outer loop). Each outer training set was further split into five subsets, applying one subset for model selection and the remaining four subsets for training (inner loop). In the inner loop, five models were developed using the inner training set and evaluated using the inner validation set, and the best-performing model was selected. Finally, the selected model was evaluated using the outer validation set, and the best model and parameters were selected.

The number of folds was determined to be balanced between maximizing the number of iterations and maintaining the number of samples in a fold not too small.

Supplementary References

1 Tofler, G. H. *et al.* Triggers and Timing of Acute Coronary Syndromes. *Am J Cardiol* **119**, 1560-1565 (2017).

2 Willich, S. N. *et al.* Weekly variation of acute myocardial infarction. Increased Monday risk in the working population. *Circulation* **90**, 87-93 (1994).

3 Ezekowitz, J. A. *et al.* The relationship between meteorological conditions and index acute coronary events in a global clinical trial. *Int J Cardiol* **168**, 2315-2321 (2013).

4 Varma, S. & Simon, R. Bias in error estimation when using cross-validation for model selection. *BMC Bioinformatics* **7**, 91 (2006).

5 Vabalas, A., Gowen, E., Poliakoff, E. & Casson, A. J. Machine learning algorithm validation with a limited sample size. *PLoS One* **14**, e0224365 (2019).
